# Supplementary material for: Perceptions of carbon dioxide emission reductions and future warming among climate experts
Source: Commun Earth Environ. 2024 Sep 12;5(1):498. doi: 10.1038/s43247-024-01661-8 (PMC11392805; doi:10.1038/s43247-024-01661-8)
Supplement: Supplementary file 2 — Supplementary Material [file 43247_2024_1661_MOESM2_ESM.pdf]

Perceptions of carbon dioxide emission reductions and future warming among climate experts

Supplementary Information

Seth Wynes[1,2*], Steven J Davis[3], Mitchell Dickau[1], Susan Ly[1], Edward Maibach[4], Joeri Rogelj[5,6], Kirsten Zickfeld[7], H. Damon Matthews[1]

*Corresponding author: swynes@uwaterloo.ca
[1] Department of Geography, Planning & Environment, Concordia University, Montréal, QC, Canada
[2] Department of Geography and Environmental Management, University of Waterloo, Waterloo, Canada
[3] Department of Earth System Science, University of California, Irvine, California, United States
[4] Communication, George Mason University, Fairfax, Virginia, United States
[5] Grantham Institute Climate Change and Environment, Centre for Environmental Policy, Imperial College London, London, UK
[6] Energy Climate and Environment Programme, International Institute for Applied Systems Analysis, Laxenburg, Austria
[7] Department of Geography, Simon Fraser University, Burnaby, British Columbia, Canada

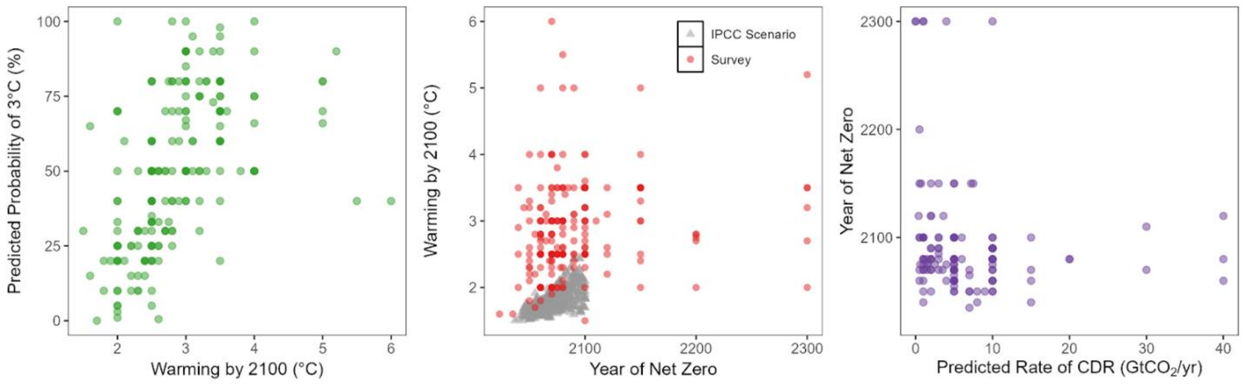

Supplementary Fig. 1: A) Plot of participant estimates of peak warming by 2100 versus probability of reaching 3°C based on participant responses. B) Year of Net Zero vs Peak Warming by 2100. Triangles represent AR6 IPCC scenarios that passed vetting (were consistent with historical trends) with peak warming indicating Median peak warming in MAGICCv7.5.3[1], whereas circles represent participant responses. C) Rate of CDR versus year of net zero for participant responses

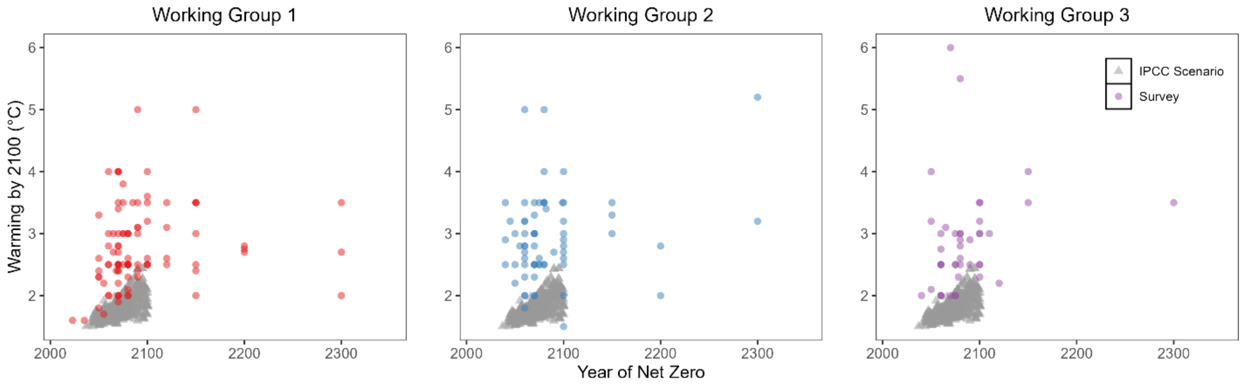

Supplementary Fig. 2: Shows results from Supplementary Fig 1B broken down by Working Group.

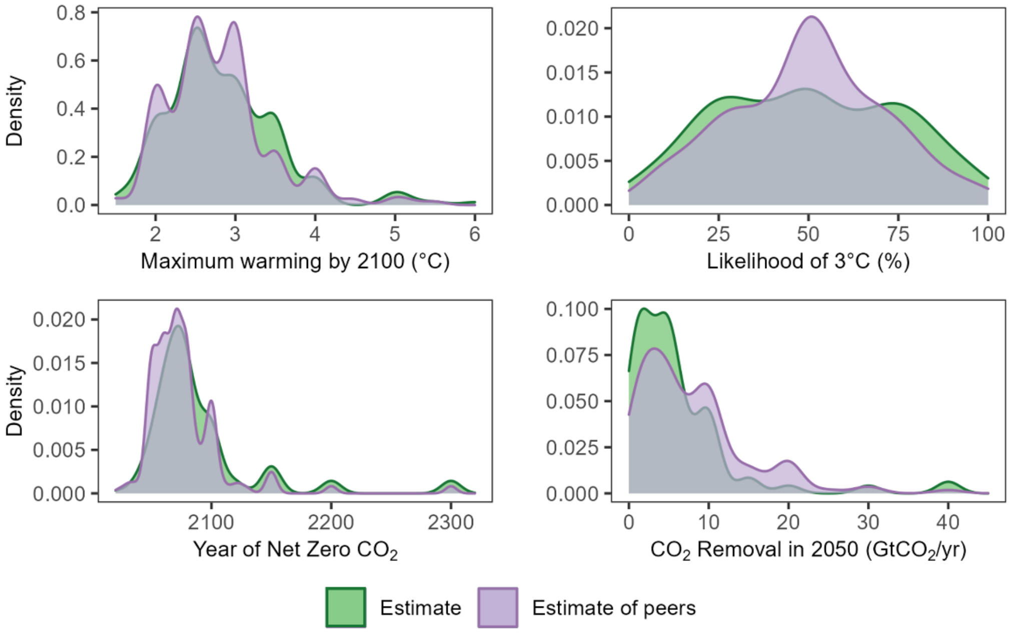

Supplementary Fig. 3: Density plots showing first-order versus second-order beliefs on future climate outcomes.

Supplementary Table 1

Continent	Total in sample	Percentage in sample	Percentage in Population
Africa	12	6	20
Asia	21	10	9
Europe	86	41	34
North America, Central America and Caribbean	55	26	19
South-West Pacific	21	10	10
South America	16	8	8

Gender			
Female	48	23	32
Male	163	77	68
Report*			
AR6-WG1	78	37	20
AR6-WG2	68	32	29
AR6-WG3	42	20	21
AR6-SYR	14	7	3
SR15	29	14	8
SROCC	29	14	9
SRCCL	25	12	9

*Note that some authors contributed to more than one report

Supplementary Table 2: Mean and unweighted means of future climate outcome estimates

Future climate outcome estimates	Mean	Weighted mean
Maximum Temperature by 2100	2.9	2.9
Likelihood of 3°C	50.2	51.4
Year of Net Zero CO_2	2090	2088
Rate of CDR	6.2	5.9
**Peer estimates**	**Mean**	**Weighted Mean**
Maximum Temperature by 2100	2.8	2.8
Likelihood of 3°C	49.8	50.1
Year of Net Zero CO_2	2077	2075
Rate of CDR	8.0	7.4

Supplementary Table 3: Spearman correlation between first and second order beliefs

	Working Group 1	Working Group 2	Working Group 3
Maximum Temperature by 2100	0.63	0.67	0.50
Likelihood of 3°C	0.71	0.72	0.63
Year of Net Zero CO_2	0.52	0.64	0.48
Rate of CDR	0.78	0.80	0.72

Note that all correlation coefficients are associated with a p-value <0.001

Supplementary Table 4: Median and IQR for estimates for future climate outcomes by continent

Continent	N	Maximum Temperature by 2100 (°C)	Likelihood of 3°C (%)	Year of net zero	Rate of CDR ($GtCO_2$)
Africa	12	2.5 (0.85)	50 (40)	2065 (30)	1.8 (3.125)
Asia	21	2.5 (1)	50 (50)	2070 (32.5)	8 (5)
Europe	86	2.5 (0.8)	40 (35)	2080 (29.5)	5 (5)
North/Central America/Caribbean	55	3 (0.85)	65 (40)	2080 (30)	3 (4)
South America	16	2.5 (0.425)	50 (50)	2070 (22.5)	5 (6.5)
South-West Pacific	21	3 (0.925)	50 (30)	2070 (40)	5 (6.4)

Note: Interquartile ranges in parentheses

Supplementary Results

Spearman rank correlations were used to test if the evidence in favor of H1 was sensitive to outliers. In the cleaned data (outliers removed) the correlation between self and peer predictions was strong for maximum warming by 2100 (r_s=.62, p<.001), strong for likelihood of 3°C (r_s=.72, p<.001), moderate for year of net zero (r_s=.55, p<0.001), and strong for rate of CDR in 2050 (r_s=.78, p<.001). With outliers retained in the dataset the correlation between self and peer predictions was strong for maximum warming by 2100 (r_s=.61, p<.001), strong for likelihood of 3°C (r_s=.72, p<.001), moderate for year of net zero (r_s=.55, p<0.001), and strong for rate of CDR in 2050 (r_s=.81, p<.001).

Kruskal-Wallis tests were used to evaluate differences between working groups (H2). In the cleaned dataset (outliers removed) there was a significant difference for the estimates of probability of reaching 3°C (H=10.4, p=.006) between the groups (WG1 med=50, WG2 med=60, WG3 med=40) with a Bonferonni-adjusted pairwise Wilcox test specifically showing differences between WG2 and WG3 (p=.02). Differences between working groups for maximum warming by 2100 (H=3.03, p=.22), year of net zero (H=1.46, p=.482), and rate of CDR in 2050 (H=0.31, p=.857) were not significant. With outliers retained in the dataset there was still a significant difference for the estimates of likelihood of reaching 3°C (H=9.89, p=.007) between the groups (WG1 med=50, WG2 med=60, WG3 med=40) with a Bonferonni-adjusted pairwise Wilcox test specifically showing differences between WG2 and WG3 (p=.02). Differences between working groups for maximum warming by 2100 (H=2.93, p=.23), year of net zero (H=1.15, p=.56), and rate of CDR in 2050 (H=0.33, p=.85) were not significant.

Supplementary Text

Page 1

Survey Title: Perceptions of future climate outcomes among IPCC researchers

Consent

You are invited to participate in a research study. Below we provide information about what participating would mean. Please read carefully before deciding if you want to participate or not. If there is anything you do not understand, or if you want more information, please ask the researcher.

A. PURPOSE

The purpose of the research is to gain a better understanding of how climate experts view future climate outcomes and evaluating their levels of optimism towards reaching key international goals.

B. PROCEDURES

If you participate, you will be asked to take an eight minute survey (with potential follow-up surveys in future years)

C. RISKS AND BENEFITS

There are no individual benefits to participating. There are no risks beyond those experienced in everyday life.

D. CONFIDENTIALITY

We will gather the following information as part of this research:

Your opinions on future climate outcomes and basic demographic information. Participants may choose to provide email addresses to receive prizes and to participate in future rounds of the survey in later years.

While we are analyzing the data, we will not allow anyone to access the information except people directly involved in conducting the research. We will only use the information for the purposes of the research described in this form.

The information gathered will be anonymized. You may choose to provide an email address so that you can be potentially contacted for follow-up waves in later years. Email addresses will be stored in a separate file with an anonymized respondent ID code but will not be saved in the same file where analysis is performed.

We will protect the information by storing the data on password protected files until the analysis is complete. We intend to publish the results of the research. Anonymized data will be made openly accessible to the public once it is published, however, it will not be possible to identify you in the published results.

F. CONDITIONS OF PARTICIPATION

You do not have to participate in this research. It is purely your decision. If you do participate, you can stop at any time. There will be no negative consequences for not participating or stopping in the middle. For those participants who provide contact information, data can be withdrawn up to two weeks following submission of the survey by emailing the researcher and asking to withdraw.

Participants will be asked to estimate the responses of their peers on certain questions. The five participants with the highest average rank on these questions will each be able to donate $100 to a recognized environmental charity of their preference. All other participants will be able to donate $2 to a charity of their choosing (from a pre-selected list of environmental charities).

G. QUESTIONS

If you have questions about the scientific or scholarly aspects of this research, please contact the researcher at: christopherseth.wynes@concordia.ca

If you have concerns about ethical issues in this research, please contact the Manager, Research Ethics,

Concordia University, 514.848.2424 ex. 7481 or oor.ethics@concordia.ca.

PARTICIPANT'S DECLARATION

By clicking "Yes" below you agree that you have read and understood this form. You also agree that you have had the chance to ask questions and any questions have been answered and agree to participate in this research under the conditions described.

Do you agree to participate?

Yes

No

Page 2

1. What do you think is the likelihood that Earth will experience global warming of 3°C or more (as compared to an 1850-1900 baseline) by or before 2100?

___%

2. We want you to think about how other IPCC authors* will answer this question. What do you think their average response will be on the previous question?

___%

*Footnote: *IPCC authors contacted for this survey include authors from the SR1.5 report and onwards.*

Page 3

3. Human-caused global warming reached 1.2°C in 2022 (as compared to an 1850-1900 baseline). Please provide your best estimate of maximum warming before or by the year 2100, in degrees Celsius.

_____°C

4. We want you to think about how other IPCC authors* will answer this question. What do you think their average response will be on the previous question?

_____°C

*Footnote: *IPCC authors contacted for this survey include authors from the SR1.5 report and onwards.*

3. If your answers to Questions 3 and 4 differed, why do you believe your peers' assessments, on average, will be different than yours?

____

Page 4

6. In what year do you think human CO2 emissions will reach net zero globally?

_____

7. We want you to think about how other IPCC authors will answer this question. What do you think their average response will be on the previous question?

_____

*Footnote: *IPCC authors contacted for this survey include authors from the SR1.5 report and onwards.*

Page 5

8. Net-zero CO2 emissions implies that any remaining CO2 emissions are matched by an equal amount of carbon dioxide removal. What do you think is the most likely rate of carbon dioxide removal* in 2050? We strongly encourage you to give your best guess, but if you are unable to answer this question, please click “Next”.

______ $GtCO_2$/yr

*Footnote: *Carbon dioxide removal: Anthropogenic activities removing carbon dioxide (CO2) from the atmosphere and durably storing it in geological, terrestrial, or ocean reservoirs, or in products. It includes existing and potential anthropogenic enhancement of biological or geochemical CO2 sinks and direct air carbon dioxide capture and storage (DACCS), but excludes natural CO2 uptake not directly caused by human activities (IPCC WG3 Annex)*

9. We want you to think about how other IPCC authors* will answer this question. What do you think their average response will be to the previous question?

______ $GtCO_2$/yr

*Footnote: *IPCC authors contacted for this survey include authors from the SR1.5 report and onwards.*

Demographics

10. Which IPCC report(s) have you contributed to? Please select all that apply.

□ AR6 WG1

□ AR6 WG2

□ AR6 WG3

□ AR6 SYR

□ SR 1.5

□ SROCC

□ SRCCL

□ Older reports not listed here

11. What chapter in AR6 best represents your central area of expertise?

[Drop down menu]

WG1 - CHPT2: CHANGING STATE OF THE CLIMATE SYSTEM

WG1 - CHPT3: HUMAN INFLUENCE ON THE CLIMATE SYSTEM

WG1 - CHPT4: FUTURE GLOBAL CLIMATE: SCENARIO-BASED PROJECTIONS AND NEAR-TERM INFORMATION

WG1 - CHPT5: GLOBAL CARBON AND OTHER BIOGEOCHEMICAL CYCLES AND FEEDBACKS

WG1 - CHPT6: SHORT-LIVED CLIMATE FORCERS

WG1 - CHPT7: THE EARTH'S ENERGY BUDGET, CLIMATE FEEDBACK, AND CLIMATE SENSITIVITY

WG1 - CHPT8: WATER CYCLE CHANGES

WG1 - CHPT9: OCEAN, CRYOSPHERE, AND SEA LEVEL CHANGE

WG1 - CHPT10: LINKING GLOBAL TO REGIONAL CLIMATE CHANGE

WG1 - CHPT11: WEATHER AND CLIMATE EXTREME EVENTS IN A CHANGING CLIMATE

WG1 - CHPT12: CLIMATE CHANGE INFORMATION FOR REGIONAL IMPACT AND FOR RISK ASSESSMENT

WG2 - CHPT2: TERRESTRIAL AND FRESHWATER ECOSYSTEMS AND THEIR SERVICES

WG2 - CHPT3: OCEAN AND COASTAL ECOSYSTEMS AND THEIR SERVICES

WG2 - CHPT4: WATER

WG2 - CHPT5: FOOD, FIBRE, AND OTHER ECOSYSTEM PRODUCTS

WG2 - CHPT6: CITIES, SETTLEMENTS AND KEY INFRASTRUCTURE

WG2 - CHPT7: HEALTH, WELLBEING AND THE CHANGING STRUCTURE OF COMMUNITIES

WG2 - CHPT8: POVERTY, LIVELIHOODS AND SUSTAINABLE DEVELOPMENT

WG2 - CHPT16: KEY RISKS ACROSS SECTORS AND REGIONS

WG2 - CHPT17: DECISION-MAKING OPTIONS FOR MANAGING RISK

WG2 - CHPT18: CLIMATE RESILIENT DEVELOPMENT PATHWAYS

WG2 - CROSS-CHPT: BIODIVERSITY HOTSPOTS

WG2 - CROSS-CHPT: CITIES AND SETTLEMENTS BY THE SEA

WG2 - CROSS-CHPT: DESERTS, SEMI-ARID AREAS, AND DESERTIFICATION

WG2 - CROSS-CHPT: MEDITERRANEAN REGION

WG2 - CROSS-CHPT: MOUNTAINS

WG2 - CROSS-CHPT: POLAR REGIONS

WG2 - CROSS-CHPT: TROPICAL FORESTS

WG3 - CHPT2: EMISSION TRENDS AND DRIVERS

WG3 - CHPT3: MITIGATION PATHWAYS COMPATIBLE WITH LONG-TERM GOALS

WG3 - CHPT4: MITIGATION AND DEVELOPMENT PATHWAYS IN THE NEAR-TO MID-TERM

WG3 - CHPT5: DEMAND, SERVICES AND SOCIAL ASPECTS OF MITIGATION

WG3 - CHPT6: ENERGY SYSTEMS

WG3 - CHPT7: AGRICULTURE, FORESTRY, AND OTHER LAND USES (AFOLU)

WG3 - CHPT8: URBAN SYSTEMS AND OTHER SETTLEMENTS

WG3 - CHPT9: BUILDINGS

WG3 - CHPT10: TRANSPORT

WG3 - CHPT11: INDUSTRY

WG3 - CHPT12: CROSS SECTORAL PERSPECTIVES

WG3 - CHPT13: NATIONAL AND SUB-NATIONAL POLICIES AND INSTITUTIONS

WG3 - CHPT14: INTERNATIONAL COOPERATION

WG3 - CHPT15: INVESTMENT AND FINANCE

WG3 - CHPT16: INNOVATION, TECHNOLOGY DEVELOPMENT AND TRANSFER

WG3 - CHPT17: ACCELERATING THE TRANSITION IN THE CONTEXT OF SUSTAINABLE DEVELOPMENT

12. What is your gender?

□ Male

□ Female

□ Non-binary

Other _________

13. What continent is your nation of citizenship located in?

□ Africa

□ Asia

□ Europe

□ North America, Central America and Caribbean

□ South America

□ South-West Pacific

Page 7

14. Please select your preferred charity from the following list.

□ **Wildlife Conservation Society** (conserving the world's wild places)

□ **Practical Action** (helping communities adapt to flooding)

□ **350.org** (international movement working to end fossil fuels)

□ **Greenpeace** (exposing environmental destruction)

□ **Oceana** (protecting and restoring the world's oceans)

□ **Cool Earth** (people-powered solutions for deforestation)

□ **Clean Air Task Force** (advancing decarbonization policies and technologies)

□ **Union of Concerned Scientists** (Scientists advocating for a sustainable future)

□ **Carbon 180** (Championing equitable carbon removal solutions)

□ **Earth Justice** (Fighting for green groups in court)

□ **Jo-Jikum** (empower young Marshall Islanders to respond to climate change)

□ **La Via Campesina** (grassroots movement for sustainable agriculture)

Page 8

15. Longitudinal data are extremely valuable to researchers who are tracking trends over time. We would like to conduct this survey again in the future, and link responses in future years between the same participants. To do this, we need personalized information from each respondent.

Please consider providing your email address so that we can track individual responses over time. This will be kept strictly confidential.

To make sure your responses are anonymous, when analyzing the data, individual emails will be replaced with an identifying code, and a key containing both the codes and emails addresses will be stored in a separate password protected file.

Email address ___________________________

□ I would like to receive feedback on results and the accuracy of my responses

Skip

Page 9

Thank you for participating in the survey!

Your participation will help us and other researchers understand the key beliefs of IPCC authors.

We are very grateful for your time!

If you have any further comments we would be happy to hear them.

Comment:

Supplementary References

1 Meinshausen, M., Raper, S. C. B. & Wigley, T. M. L. Emulating coupled atmosphere-ocean and carbon cycle models with a simpler model, MAGICC6 – Part 1: Model description and calibration. *Atmos. Chem. Phys.* **11**, 1417-1456, doi:10.5194/acp-11-1417-2011 (2011).
